# Drivers of inappropriate antibiotic use in low- and middle-income countries

**DOI:** 10.1093/jacamr/dlad062

**Published:** 2023-05-31

**Authors:** Idemudia Imonikhe Otaigbe, Charles John Elikwu

**Affiliations:** Department of Medical Microbiology, School of Basic Clinical Sciences, Benjamin Carson (Snr.) College of Health & Medical Sciences, Babcock University, Ilishan Remo, Ogun State, Nigeria; Department of Medical Microbiology, School of Basic Clinical Sciences, Benjamin Carson (Snr.) College of Health & Medical Sciences, Babcock University, Ilishan Remo, Ogun State, Nigeria

## Abstract

Antimicrobial resistance (AMR) is a global security threat that accounts for about 700 000 deaths annually. Studies have shown that antimicrobial resistance could result in a 2% to 3.5% reduction in global Gross Domestic Product by 2050 and a loss of between 60 and 100 trillion US dollars, worth of economic output resulting in significant and widespread human suffering. Low- and middle-income countries (LMICs) will be worse hit by an unchecked rise of AMR. For example, it is predicted that AMR could kill about 4.1 million people in Africa by 2050 if it is not curbed. Similarly rising rates of AMR will lead to increased treatment costs and an inability to attain universal health coverage, in LMICs with fragile health systems. Sadly, AMR is driven by the inappropriate use of antimicrobials, especially antibiotics. Inappropriate antibiotic use is a pertinent problem in LMICs where regulatory frame works are weak. Inappropriate antibiotic use in LMICs is a multifaceted problem that cuts across clinical and veterinary medicine and agriculture. Therefore, efforts geared at curbing inappropriate antibiotic use in LMICs must identify the factors that drive this problem (i.e. inappropriate antibiotic use) in these countries. A clear knowledge of these factors will guide effective policy and decision making to curb inappropriate antibiotic use and ultimately AMR. The focus of this review is to discuss the factors that drive inappropriate antibiotic use in LMICs.

## Introduction

Antimicrobial resistance (AMR) is a global security threat.^1^ It is driven by the inappropriate use of antimicrobials, particularly antibiotics, in humans, animals and veterinary medicine.^1^ The inappropriate use of antibiotics also exacts adverse clinical and economic effects on patients.^2,3^ The adverse economic effects include increased costs of healthcare borne by patients, whereas the adverse clinical effects include increased consumption of antibiotics, increased length of hospital stay, morbidity, mortality and certainly the emergence of AMR.^2,3^

AMR is considered one of the biggest threats to global health, food security and development.^4^ AMR accounts for an estimated 700 000 deaths per year globally.^4^ If AMR is not curbed, it could result in about 10 million global deaths per year and 4.1 million deaths in Africa by 2050.^5,6^ AMR could also have devastating economic impacts as it could result in a 2% to 3.5% reduction in global Gross Domestic Product by 2050, a loss of between 60 and 100 trillion US dollars (US$) of economic output, increased treatment costs (resulting in increased morbidity and mortality in poorly funded fragile health systems in many LMICs) and a failure to achieve universal health coverage and the Sustainable Development Goals (SDGs), particularly in LMICs.^4,7^ Increased morbidity and mortality (arising from rising rates of AMR) could result in declining labour supply and an additional 28.3 million people being pushed into extreme poverty by 2050 due to high costs of treatment and chronic infections.^7^ AMR related morbidity and mortality in animal husbandry could affect the livelihood of farmers and also threaten food security.^7^

Another danger of AMR is that it results in market failures, low return on investment and disinclination to invest in research and development in new antimicrobial agents, particularly antibiotics.^8^ Rather than invest in developing new antibiotics, multinational pharmaceutical companies are choosing the more profitable option of developing drugs for non-communicable diseases.^8^ Also, the few new antibiotics being developed are expensive and show little benefit compared to existing antibiotics.^9^ For example, the World Health Organization notes that only 2 out of the 12 antibiotics approved since 2017 represent a new class of antibiotics.^9^ The remainder are derivatives of already existing antibiotics.^9^ Citizens of LMICs would have to bear the unduly high costs of purchasing these newly developed antibiotics since existing and cheaper antibiotics are ineffective.^1^ Sadly, poverty and other factors such as weak antibiotic supply chain management systems deny many citizens of LMICs from accessing both old and new antibiotics.^1^

Globally, the inappropriate use of antibiotics in veterinary medicine and agriculture compounds the problem of AMR as it results in the emergence of antibiotic-resistant bacteria and resistance genes, which can be transmitted to humans through the food chain or through direct contact with animals.^10,11^ However, in LMICs, the inappropriate use of antibiotics in agriculture is exacerbated by weak or non-existent regulatory frameworks.^10,11^

The COVID 19 pandemic has aggravated the global problem of inappropriate antibiotic use.^12^ A lack of definite treatment protocols fuelled the irrational prescription and use of antibiotics for COVID 19 patients.^12^ The danger of such irrational use of antibiotics is the future emergence of drug resistant bacteria in the community and healthcare settings.^12^

Although inappropriate antibiotic use is a global problem, the political and socio-cultural peculiarities in many LMICs make it a multifaceted problem.^13–15^ Therefore, policy formulation and other efforts to curb inappropriate antibiotic use (and AMR) in LMICs must have a precise knowledge of the specific drivers of inappropriate antibiotic use in LMICs.^13–15^ The focus of this review is to discuss the drivers of inappropriate antibiotic use in LMICs.

## Defining inappropriate antibiotic use

It is important to clearly define the concept of inappropriate antibiotic use to have a precise understanding of the factors that drive inappropriate antibiotic use in LMICs. The term inappropriate antibiotic use implies that antibiotics are used in an inappropriate manner or injudiciously.^16^ However, a robust definition of inappropriate antibiotic use should be comprehensive, as clearly shown in the descriptions below. In this regard antibiotic use is deemed inappropriate if any of the following scenarios have occurred:

1. **Inappropriate indication:** this implies that antibiotics are prescribed or consumed when not indicated.^17^ It also includes prescribing antibiotics for prophylaxis even when available guidelines clearly obviate the need for prophylaxis.^17^
2. **Inappropriate choice or selection:** examples of wrong antibiotic choices include choosing antibiotics that are not recommended in guidelines and using antibiotics that do not cover the microbial spectrum or that lack activity at the target organ or system.^16,18^ The concept of inappropriate choice may also be contextual and may include the administration of antibiotics to patients in the light of clear contraindications, risks of adverse events or other patient specific issues that make the choice of a particular antibiotic inappropriate.^18,19^
3. **Inappropriate quality:** this refers to the consumption of sub-standard, falsified or expired antibiotics. The use of sub-standard antibiotics results in sub-optimal inhibitory concentrations of the active pharmaceutical ingredient, treatment failures and the emergence of antimicrobial resistance.^20,21^
4. **Inappropriate dosing**: administering sub-optimal doses of antibiotics (e.g. wrong dosage, timing, route of administration and duration of therapy) are examples of inappropriate antibiotic use.^16,17^
5. **Inappropriate dispensing**: guidelines for appropriate dispensing emphasize that prescribed antibiotics should be dispensed by qualified and licensed dispensers.^22,23^ At the point of dispensing, patients should be provided appropriate information regarding the antibiotic, e.g. dosage, possible adverse effects, the need to adhere to the prescription etc.^22,23^ When antibiotics are not appropriately dispensed then patients are put at the risk of inappropriate antibiotic use and associated adverse outcomes.^22,23^
6. **Lack of adherence of the patient to antibiotic prescriptions:** Some patients may not adhere strictly to antibiotic prescriptions due to inadequate information at the point of dispensing or financial constraints (which may hinder some patients from completing doses of unaffordable or expensive antibiotics).^24,25^

## Factors driving inappropriate antibiotic use in low- and middle-income countries (LMICs)

Several factors drive inappropriate antibiotic use in LMICs. Some factors are peculiar to LMICs while other factors are common to all countries (i.e. high-, LMICs). This review draws attention to those factors that are peculiar to LMICs. Factors that are common to all countries irrespective of their income categorizations are discussed later in this review. The factors that drive inappropriate antibiotic use in LMICs include:

### Lack of political will by LMIC governments

The term ‘political will’ is defined as ‘the commitment of political leaders and bureaucrats to undertake actions to achieve a set of objectives and to sustain the costs of those actions over time’.^26^ The political will of an incumbent government is a strong factor that influences decision making, budgetary allocations, public private partnerships and the inflow of technical expertise and/or funds into a nation.^27^ Political will is a key element required to curb inappropriate antibiotic use in LMICs as it fosters the development and implementation of National Action Plans (NAPs) on AMR.^28^ The dearth of political will in LMICs results in poor or absent regulatory frameworks to curb inappropriate antibiotic use and AMR.^28^ The major factor impeding the development and implementation of NAPs in LMICs is the dearth of political will.^28^

### Poor implementation of National Action Plans on AMR

In addition to poor political will, some other factors that impede the implementation of NAPs on AMR include:

**1. Insufficient funds**: Developing and implementing NAPs involves a wide range of cost intensive, multidisciplinary activities.^28^ Many LMICs, however, lack the financial resources required to implement NAPs.^28^ For example, in 2017, Zimbabwe’s 5-year NAP on AMR was estimated to cost about US$44.6 million.^28,29^ This amount of money is clearly expensive for many LMICs.^28^
**2. A lack of multi-sectoral coordination:** The implementation of NAPs is also hindered by poor multi-sectoral coordination in LMICs.^28,30^ The problem of inappropriate antibiotic use involves diverse stakeholders, and traverses ecological and geographical boundaries.^30^ Since many LMICs lack policies and frameworks required to support multi-sectoral coordination, efforts to curb inappropriate antibiotic use and AMR usually occur in a fragmented, uncoordinated and non-inclusive manner.^28,30^ For example, in some LMICs the Ministry of Finance (which is responsible for budgetary allocations) is not involved in discussions regarding the development or implementation of NAPs.^28^
**3. A paucity of data and weak surveillance structures:** Data collection in many LMICs is manual and usually consists of incomplete data sets, which fail to synergize laboratory data (e.g. antibiotic susceptibility patterns) with clinical data.^31^ This results in poor surveillance of local antibiotic consumption and resistance rates.^31^ In the absence of data and effective surveillance structures, such information is not readily available to guide antibiotic therapy at institutional levels, nor policy formulation at national levels.^31^
**4. A dearth of technical capacity:** many LMICs lack personnel with adequate training in antimicrobial stewardship and other skills required for effective networking, communication and governance of initiatives to curb inappropriate antibiotic use and AMR.^30^

### Lack of access to quality laboratory services

A major challenge in LMICs is the lack of access to quality laboratory services, which results in diagnostic uncertainty, poor clinical decision making, inappropriate antibiotic use and the emergence of AMR.^32^ Lack of access to quality laboratory services in LMICs may be regarded from two perspectives: inadequate access and inequitable access.^33–36^

**Inadequate access:** this refers to an insufficient quantity and quality of laboratory services in many LMICs.^33,34^ A study showed that only 380 laboratories in Sub-Saharan Africa were accredited to international standards and that 37 of the 49 countries surveyed had no laboratories accredited to international quality standards.^35^ In many LMICs, laboratories are challenged by deficiencies in the quality of services they render.^33,36^ Examples of these deficiencies include lack of qualified manpower, inadequate infrastructure, insufficient (or non-existent) laboratory supplies, deficits in supply chain management, poor equipment maintenance, inadequate quality management systems and a dearth of regulation by government.^33,36^ In addition, pathogen detection and antibiotic susceptibility testing involve the use of obsolete phenotypic methods that are often slow, unstandardized and prone to errors, and therefore result in inaccurate clinical decision making.^37^

**Inequitable access**: this refers to the uneven distribution of quality laboratory services in LMICs.^35^ The previously mentioned study showed that 91% of internationally accredited laboratories in Africa were in South Africa.^35^ Also, many rural communities in LMICs lack laboratory services because services in LMICs are usually located in urban areas.^38,39^ In the absence of guidance, by diagnostic laboratory services, doctors practising in rural areas usually resort to presumptive management of patients, blind prescription of antibiotics or referral of very sick cases.^38,39^ This results in significant morbidity and mortality among these patients.^38,39^

### Poor use of laboratory services by clinicians

Another driver of inappropriate antibiotic use in LMICs, is the poor use of laboratory services by clinicians due to the following reasons: the wrong assumption that clinical diagnosis is sufficient and that laboratories are not necessary to manage patients with infections^40,41^; subservience to hierarchy (e.g. a senior physician instructs a junior physician not to use the laboratory)^40,41^; poor communication between physicians and the hospital’s laboratory^40,41^; a lack of awareness of the problem of inappropriate antibiotic use and the role of the clinical microbiology laboratory in curbing AMR.^40,41^ Other reasons include long turn-around times or delays in sending out results to clinicians (these delays subsequently render such results irrelevant to patient management) ^40,41^; lack of confidence in the accuracy of results; absence of clinical microbiologists^40,41^ and the patients not able to afford laboratory tests.^40,41^ When physicians fail to use clinical microbiology laboratory services, they are usually left with the option of blind prescription of broad-spectrum antibiotics.^40,41^ Subsequently, when treatment failure occurs, physicians resort to repeated (or ‘trial and error’) antibiotic switches that result in patients bearing the adverse clinical and economic consequences of inappropriate antibiotic use.^40,41^

## Poor Infection Prevention and Control (IPC) protocols in the community and healthcare setting

Poor IPC protocols in the community and healthcare setting may be viewed from three perspectives^42–45^:

1. Lack of access to WASH (water, sanitation and hygiene) facilities in communities

In many LMICs, lack of access to WASH facilities results in a vicious cycle of high burdens of communicable diseases, increased antibiotic consumption and the emergence of AMR.^42,43^ According to a 2021 report, approximately 48 million Nigerians still defecate in the open, whereas only 8% of the population practices safe hand washing.^46^ The same report also showed that 23% of Nigerians lack access to basic water supply services and only 10% of the population had access to basic water, sanitation and hygiene services combined.^46^ Unfortunately, available data show that diarrhoeal diseases disproportionately affect places with inadequate access to WASH facilities, as well as low-income or marginalized populations.^47^ For example, in 2016, diarrhoea was the eighth leading cause of death, responsible for more than 1.6 million deaths, globally.^48^ More than a quarter (26.93%) of these diarrhoeal deaths occurred among children younger than 5 years of age, and about 90% of these diarrhoeal deaths occurred in south Asia and sub-Saharan Africa.^48^ High diarrhoeal burdens also result in increased consumption of antibiotics.^43^ For example about 494 million cases of diarrhoea are treated with antibiotics each year in Brazil, Indonesia, India and Nigeria alone.^43^ Such high antibiotic consumption rates are drivers of AMR in these countries and other LMICs in which antibiotics are used inappropriately.^43^

2. Poor vaccine coverage and uptake in communities

Vaccination has a positive impact on population health, productivity and education and adds value by reducing disease burden on individuals, families and communities.^49^ It is estimated that vaccination yields a return on investment valued at about US$44 for every dollar invested.^50^ By preventing the occurrence or spread of bacterial infections, vaccination reduces antibiotic consumption and the emergence of AMR.^51^ However, one in five African children and one in four children in South America do not receive all required childhood immunizations resulting in the occurrence of vaccine-preventable diseases and deaths.^49,52^ In Africa, more than 30 million children under five still suffer from vaccine-preventable diseases and of these number of children, over half a million die annually, representing approximately 58% of global deaths due to vaccine-preventable diseases.^49,53^ Also, poor vaccine uptake in LMICs results in increased consumption of antibiotics, increased inappropriate antibiotic use and the eventual emergence of AMR.^54^

Factors responsible for low vaccine coverage include distrust of parents and caregivers in vaccination, considerable distance between the home and the vaccination centre, language barriers and inadequate infrastructures to maintain the cold chain and adequate supply of vaccines.^44^ The COVID 19 pandemic has further worsened this picture.^55^ Before the beginning of the COVID 19 pandemic, in 2019 about 19 million children under the age of 1 year failed to receive basic vaccines through routine childhood immunization programmes.^55^ However, by 2021 this figure had risen to about 25 million children under the age of 1 year. In addition, 18 million children who have never received any routine childhood vaccine doses (i.e. zero-dose children) did not receive any vaccines in 2021.^55^ This is the highest number of poor vaccine uptake among zero-dose children since 2005.^55^ Sadly, almost all zero-dose children live in LMICs, primarily in Africa and South-East Asia.^56^

3. Poor adherence to IPC protocols in healthcare facilities

Poor adherence to IPC protocols is also a major challenge in healthcare settings in LMICs.^45^ Many healthcare facilities in LMICs lack adequate WASH facilities.^45^ This results in a vicious circle characterized by low quality of care, the occurrence of healthcare-associated infections (HCAIs), increased antibiotic consumption and significant morbidity and mortality.^45^

Also, a dearth of vaccination programmes for health workers and vaccination hesitancy among health workers exacerbates the problems posed by defective IPC protocols and HCAIs.^57,58^ When healthcare workers fail to take required vaccinations they are at considerable risk of developing vaccine-preventable diseases (including associated morbidity and mortality) and transmitting these diseases to patients.^57,58^

### Inadequately trained drug dispensers

Dispensing involves ‘the review of a prescription and the preparation, packaging, labelling, record keeping and transfer of the prescribed medicine including counselling to a patient, their agent, or another person who is responsible for the administration of the medicine to that patient’.^59^ Dispensing remains a vital element of the rational use of antibiotics and efforts to promote appropriate antibiotic use must incorporate good dispensing practices.^23^ All resources used in patient care before dispensing may be wasted if the designated patient does not receive the proper antibiotic in an effective form, in the proper packaging, at the proper dosage and advice (e.g. correct dose, possible adverse effects etc.).^22^ For proper dispensing to occur, the dispenser(s) must be well trained and have a basic knowledge about the antibiotics being dispensed. Such knowledge includes the correct dosage of the antibiotic, side effects, possible interactions with other medicines etc.^22^ In addition, the dispenser must have the right attitude and skills required to communicate effectively with patients.^22^ However, there is a dearth of trained pharmacists in many LMICs. This results in drug dispensing being done by poorly trained staff who lack the required knowledge, attitude and skills of proper drug dispensing.^60^ The lack of training therefore compromises their ability to contribute to curbing inappropriate antibiotic use and AMR in health facilities.^60^

### Illegal drug vendors in the community

Closely related to the problem of inadequately trained drug dispensers is the problem of illegal drug vendors in the community.^61^ Illegal drug vendors contribute significantly to inappropriate antibiotic use and the emergence of AMR in LMICs.^61^ Globally, over 50% of antimicrobials are purchased without prescriptions in the community.^61^ These antimicrobials are sold by illegal drug vendors in markets, stalls and commuter vehicles in many LMICs.^61^

### Lack of access to effective antibiotics

Access to effective medicines is a fundamental human right and a key component of the SDGs.^62^ It is defined as ‘having drugs continuously available and affordable at public or private health facilities or drug outlets that are within one hour’s walk of the population’.^63^

A major challenge in LMICs is a lack of access, to effective antibiotics.^22^ Several factors are responsible for a lack of access to effective antibiotics in LMICs. These factors include:

1. **Poor regulatory frameworks** that result in the manufacture, importation and distribution of sub-standard or falsified medicines.^22,64^ About 17% of these sub-standard or falsified medicines are antibiotics.^64^ Poor regulatory frameworks also result in individuals with no pharmaceutical training being involved in the importation and distribution of antibiotics in LMICs.^22^ These individuals lack basic knowledge of standard procurement and distribution practices of antibiotics (and other medicines) and therefore contribute to the problem of inappropriate antibiotic use by their distribution of sub-standard antibiotics in LMICs.^22^
2. **Poverty and inadequate funding of the health sector** deny patients from accessing quality healthcare and effective antibiotics in LMICs.^21^ Inadequate budgetary allocations result in infrastructural deficits and chronic shortages of antibiotics and other medicines that (if available) are usually sold to patients at subsidized rates in public health facilities.^21^ However, when these antibiotics are not available, patients are made to bear high medical costs when they buy antibiotics (at higher prices) from private pharmacies or drugstores.^21^ In many cases, patients cannot afford to buy these antibiotics.^21^ Subsequently, their failure to use antibiotics compromises their quality of care.
3. **The diminishing antibiotic pipeline and waning research and development efforts in new antibiotics** reduces the global portfolio of available and effective antibiotics.^8^ Even after the discovery of a new antibiotic, regulatory hurdles, weak health systems and unreliable supply chains can delay market entry of new antibiotics into LMICs.^21^

## Factors that drive inappropriate antibiotic use in all countries irrespective of income level

The factors that follow are common to all countries irrespective of income levels (i.e. high-, low- or middle-income countries). However, they are more pronounced in LMICs due to weak regulatory structures and a dearth of political will to curb inappropriate antibiotic use.^13^

These factors include:

### The perception, values and beliefs of consumers or end users of antibiotics

Consumers or end users of antibiotics include individuals, families and communities.^21^ The attitudes, beliefs and socio-cultural values of consumers influence the consumption of antibioitcs.^14,65^ Even when antibiotics are deemed unnecessary, individuals, relatives or care givers are known to demand for antibiotics.^66^ This is a common occurrence in all countries irrespective of income level.^14,15^ However, certain socio-cultural differences that are peculiar to some LMICs may accentuate this problem. For example, in LMICs with strong patrilineal, traditional or religious cultures, the decision to use antibiotics or seek healthcare is largely dependent on the husband (or male family head), the traditional ruler or religious leader.^67^ In addition, strong socio-cultural preferences for a particular route of administration may also result in inappropriate dosing.^21^ For example, it is not uncommon for outpatients in some LMICs to insist on intravenous or intramuscular antibiotics even when oral alternatives may suffice.^21^

### Physician (prescriber) factors

Several factors may influence physicians to prescribe antibiotics inappropriately.^68,69^ Examples include:

1. The physicians’ personal attributes: These include clinical experience, exposure to continuous medical education and speciality.^68,69^
2. The physician’s attitude to the problems of inappropriate antibiotic use and antibiotic resistance (see Table 1).^68–70^
3. A lack of institutional guidelines and antimicrobial stewardship programmes may also result in inappropriate antibiotic prescribing.^71^
4. Marketing and promotional activities by pharmaceutical companies may influence the prescribing patterns of physicians.^72,73^ For example, a study done in Ethiopia showed that 55.9% of physicians perceived that pharmaceutical marketing strategies influenced their prescribing behaviour.^73^ Another study done in the USA showed that pharmaceutical marketing was associated with more prescriptions per patient.^72^

**Table 1.** Physician’s attitude to prescribing antibiotics appropriately^68–70^

Physician’s attitude	Remark
Self-approval	Believes there is no need to improve his/her current prescribing practices.
Fear of adverse events	Prescribing inappropriate antibiotics due to fear of adverse outcomes, litigation, losing a patient’s patronage etc.
Apathy	The physician exhibits indifference to the problems arising from prescribing inappropriate antibiotics.
Lack of knowledge or information	Expresses ignorance about problems which may arise from prescribing inappropriate antibiotics.
Refuses to accept personal responsibility	Refuses to accept personal responsibility to prescribe antibiotics appropriately.
Independence or autonomy	Some physicians prefer certain durations or routes of administration of antibiotics in spite of contrary advice or recommendations in guidelines. The physician’s attitude or decision-making is independent or autonomous.
Diagnostic uncertainty	Prescribing antibiotics even when uncertain about the need for antimicrobials.
Pressure	Prescribing due to pressure, e.g. pressure from patients or pharmaceutical companies.

### The use of antibiotics in agriculture and veterinary medicine

In many countries, the use of antibiotics in agriculture exceeds that in human beings.^10,11^ Antibiotics are used for treatment, disease prevention or growth promotion in livestock and crops.^10,11^ The inappropriate use of antibiotics in agriculture leads to the emergence of antibiotic-resistant bacteria and resistance genes that can be passed on to humans via the food chain or by direct contact with animals.^74^ For example a study done in Vietnam among 94 aquaculture farms reported an average of 3.3 kg of antibiotics for every tonne of aquatic product, most of which were on the World Health Organization’s highly or critically important antimicrobials list.^74^ In addition the same study showed that 26.9% of fish bought from local markets tested positive for antimicrobial residues.^74^

## Recommendations

A major recommendation is to build political will to curb inappropriate antibiotic use and AMR in LMICs. Building political will can be a complex issue and individuals or groups seeking any health reform must have an understanding of their nation’s political peculiarities, the political process and the dynamics involved in getting issues on the agenda of government.^75,76^ Suggested measures to build political will include lobbying,^75^ using policy windows^77^ (a policy window is an exceptional, fleeting period of time when there is a greater likelihood of initiating policy change than usual,^77,78^ e.g. the emergence of a new Minister of Health with a keen interest in curbing AMR represents a policy window to build political will to curb AMR) and engaging policy entrepreneurs^77^ (policy entrepreneurs are individuals who engage in collaborative efforts in and around government to promote policy innovations or health reforms^79^; they could be within or outside government, in elected or appointed positions, in interest groups or research organizations^77,79^).

Also, LMIC governments must be committed to developing and implementing NAPs on AMR.^28^ These NAPs must incorporate the One Health approach of curbing AMR in humans, agriculture and the environment.^80^ Funding and technical expertise to drive the implementation of NAPs may be sought from donor organizations and global institutions that are committed to curbing inappropriate antibiotic use and AMR.^28^ LMIC governments should also develop and implement National Strategic Laboratory Plans that would holistically address deficits and gaps in laboratory capacity and better position laboratories to contribute to curbing inappropriate antibiotic use and AMR.^81^

Budgetary allocations and development aid for WASH facilities should be significantly increased^82,83^ and LMIC governments must be committed to improving vaccine uptake and coverage through increased funding for infrastructure and surveillance of vaccine-preventable diseases.^84^ Establishing IPC teams in healthcare facilities in LMICs will curb the occurrence of HCAIs and reduce antibiotic consumption.^85^ Digital technology also offers opportunities to enhance IPC in healthcare facilities in LMICs.^86^ For example, mobile apps and wearable devices (e.g. wrist bands, badges etc.) could be used to improve hand hygiene practices by giving health workers, visual or audible reminders to perform hand hygiene.^86^

Antimicrobial Stewardship (AMS) Programmes are instrumental in curbing inappropriate antibiotic use and AMR and should be implemented in healthcare settings in LMICs.^87^ However, effective AMS programmes must be integrated with other components required for health systems strengthening i.e. IPC, WASH facilities, adequate diagnostic microbiology services and efficient governance frameworks.^88^

Digital tools such as mobile apps are low-hanging fruit that could be easily deployed by AMS programmes to guide appropriate prescribing of antibiotics in healthcare settings in LMICs.^89^

Efforts should be made to increase access to antibiotics in LMICs to reduce the infectious disease burden in these countries.^1,21^ Some LMICs have efficient supply chain management systems for drugs required in the management of HIV, tuberculosis and malaria.^21,90^ Such supply chain management systems could be expanded to improve access to antibiotics. LMICs with National Health Insurance Schemes could deploy funds from these schemes to procure essential medicines (including antibiotics) and provide them at subsidized rates to patients.^21^

Increased public enlightenment and education of health workers on appropriate antibiotic use and the danger posed by AMR is also recommended.^91^ Topics such as appropriate antibiotic use and AMR should be included in the curricula of medical and agricultural schools in LMICs to sensitize students to the problems of inappropriate antibiotic use and AMR.^91^

The private sector has an immense role to play in the fight against inappropriate antibiotic use and AMR.^92–95^ The huge financial, technical and infrastructural gaps in many LMICs make it difficult for LMIC governments to effectively curb inappropriate antibiotic use and AMR.^92,95^ However, the private sector can provide critical bridge financing, technical expertise and infrastructure.^92,93^ LMIC governments should engage the private sector through public private partnerships and create a favourable investment climate (e.g. removing import barriers that raise the costs of acquiring diagnostic microbiology equipment) for private sector participation in efforts to curb inappropriate antibiotic use and AMR.^92,93,95^

## Conclusion

Rising rates of antimicrobial resistance (AMR) and waning research and development regarding new and effective antibiotics have made it imperative to curb the inappropriate use of antibiotics in LMICs. It is however, necessary to identify the factors which drive inappropriate antibiotic use in LMICs. A proper knowledge of these factors will guide policy formulation and other concerted efforts to curb this problem.

## Data Availability

There are no data in this work.
